# Commentary: Dietary methionine influences therapy in mouse cancer models and alters human metabolism

**DOI:** 10.3389/fonc.2020.01071

**Published:** 2020-07-06

**Authors:** Muhammad Abbas Abid, Muhammad Bilal Abid

**Affiliations:** ^1^Department of Otolaryngology – Head & Neck Surgery, Johns Hopkins University School of Medicine, Baltimore, MD, United States; ^2^Divisions of Hematology/Oncology & Infectious Diseases, Department of Medicine, Medical College of Wisconsin, Milwaukee, WI, United States

**Keywords:** methionine restriction, tumor metabolism, gut microbiome, dietary interventions, precision oncology

Cancer cells depend upon a continuous supply of nutrition and interrupting that supply curtails tumor growth. One-carbon metabolism pathway is central to tumor sustenance and, through its role in DNA methylation and nucleotide synthesis and repair, several chemo-radiation strategies have exploited the cycle. Solid tumors (including cancer stem cells) are auxotrophic—essential for tumor growth but cancer cells are unable to synthesize it indigenously—for methionine, an essential amino acid involved in one-carbon metabolism. At the cellular level, methionine is needed for the reduction of oxidative stress and repair of DNA damage. Exploiting this cancer vulnerability could be a druggable target to achieve effective and sustained cancer kill, in conjunction with conventional DNA-damaging therapies such as chemotherapy and radiation.

Pre-clinical and clinical studies have shown that methionine restriction ceases tumor growth and sensitizes cancers to chemotherapy, due to an S/G2 cell-cycle arrest (1–4). The synergistic effect of methionine restriction with anti-metabolite-based chemotherapy has been demonstrated in phase I and phase II clinical trials in patients predominantly with gastrointestinal tract cancers. A randomized prospective trial in gastric cancer patients receiving 5-fluorouracil (5-FU) showed that total parenteral nutrition (TPN) restricted in methionine achieved a more effective antitumor kill in comparison to TPN that contained methionine (3). Another clinical trial demonstrated the efficacy of combined dietary methionine restriction in conjunction with FOLFOX regimen in patients with metastatic colorectal cancer (4).

Gao et al. extended these findings and established a direct link between methionine restriction and cancer outcomes. In a concurrent murine and controlled human study, they demonstrated that methionine restriction impeded tumor growth in conjunction with anti-metabolite chemotherapy and radiation—cancer therapeutic modalities that exploit the same cancer metabolism pathway as methionine depletion (5).

The group first unveiled the link between methionine restriction and tumor growth retardation in two patient-derived xenograft mice models of colorectal cancer with RAS mutation. The findings were validated via metabolomic profiling. The investigators then showed that methionine restriction synergizes with 5-FU to markedly reduce tumor growth in one of the 5-FU-resistant cell lines. The group then examined the impact on a radiation-resistant soft-tissue sarcoma mouse model with compound conditional mutations in oncogenic *Kras* (activating mutation) and tumor-suppressor *p53* (inactivating mutation). They showed that methionine restriction did not inhibit tumor by itself but did so in combination with radiation. Metabolomic analysis further revealed that methionine restriction disrupted one-carbon metabolism cycle, perturbed nucleotide synthesis and markedly reduced the levels of methionine-related metabolites.

These interesting, pre-clinical findings were reproduced in a proof-of-concept clinical study. When six healthy individuals were subjected to a possibly safe methionine restricted diet, it fundamentally disrupted one-carbon metabolism and plasma methionine-related metabolites highly correlated with those found in mouse models. This study provided evidence that strategic dietary modifications could be used to inhibit tumor growth by disrupting cancer metabolism, and potentially make relapsed/refractory cancers sensitive to frontline treatments again. In another murine study, Wang et al. showed that cancer stem cells exhibit overactive methionine metabolism and an overexpression of methionine adenosyltransferase II α (MAT2A) enzyme (6).

Evolving data around tumor metabolism may potentially disentangle the controversy related to the use of TPN in terminally ill cancer patients. Albeit earlier studies showed some benefit of TPN in patients undergoing bone marrow transplantation (BMT) (7), more recent evidence suggested no benefit of TPN usage in cancer patients: no improvement in the quality of life or outcomes (8, 9). Further evidence related to cancer metabolism would greatly help clinicians during tough clinical conversations and decisions associated with TPN usage.

Even though metabolism-based diagnostic strategies are standard in cancer care, the safety and efficacy of metabolism-based therapeutics still need to be established. In contrast to other non-communicable illnesses, such as cardiovascular diseases and diabetes mellitus, where the role of metabolic interventions is a fundamental part of treatment, the role of dietary interventions in cancer still needs to be validated. Depletion of essential amino acids may have detrimental toxicities on normal cells as well. Toxicity and feasibility studies in larger clinical cohorts are awaited before any targeted metabolism therapeutics could be employed in clinical practice.

Another metabolomic-based intervention strategy that is finding its role in cancer therapeutics is the gut microbiome. Strategies are being evaluated to transform the resident gut taxa from a dysbiotic (altered) state to a symbiotic state and re-direct the adaptive immune system toward cancer cells. Resident gut taxa and its diversity impact the adaptive immune system and differentially activate either the effector T-cells or regulatory T-cells, via production of metabolites. This distinctive T-cell differentiation then either enhances or dampens responses to immunotherapy and, secondarily, survival (10).

The gut microbiome is gaining unprecedented attention due to growing evidence of its role in immunotherapy. Bacteria belonging mainly to the *Firmicutes* and *Verrucomicrobia* phyla have specifically been shown to enhance responses to immunotherapy (10, 11). Coincidently, bacteria belonging to the same phyla are associated with metabolism of dietary methionine in the human gut (12, 13). The gut microbiome manipulation holds potential in hematological malignancies as well as solid tumors and specific gut taxa—potential biomarkers of response and survival—are being identified. Once methionine depletion strategy is validated in preclinical and clinical studies, combined gut microbiome manipulation and dietary modification hold promise for a cumulative impact on tumor growth inhibition and will carry broader therapeutic applications: from immuno-oncology to secluded solid tumor microenvironment (Figure 1). Adjunctive interventions such as single- or multi-strain probiotics (a rationally-designed consortia of live bacteria), prebiotics, narrow spectrum antibiotics, and fecal microbiota transplantation may further be examined (11, 14).

**Figure 1:**
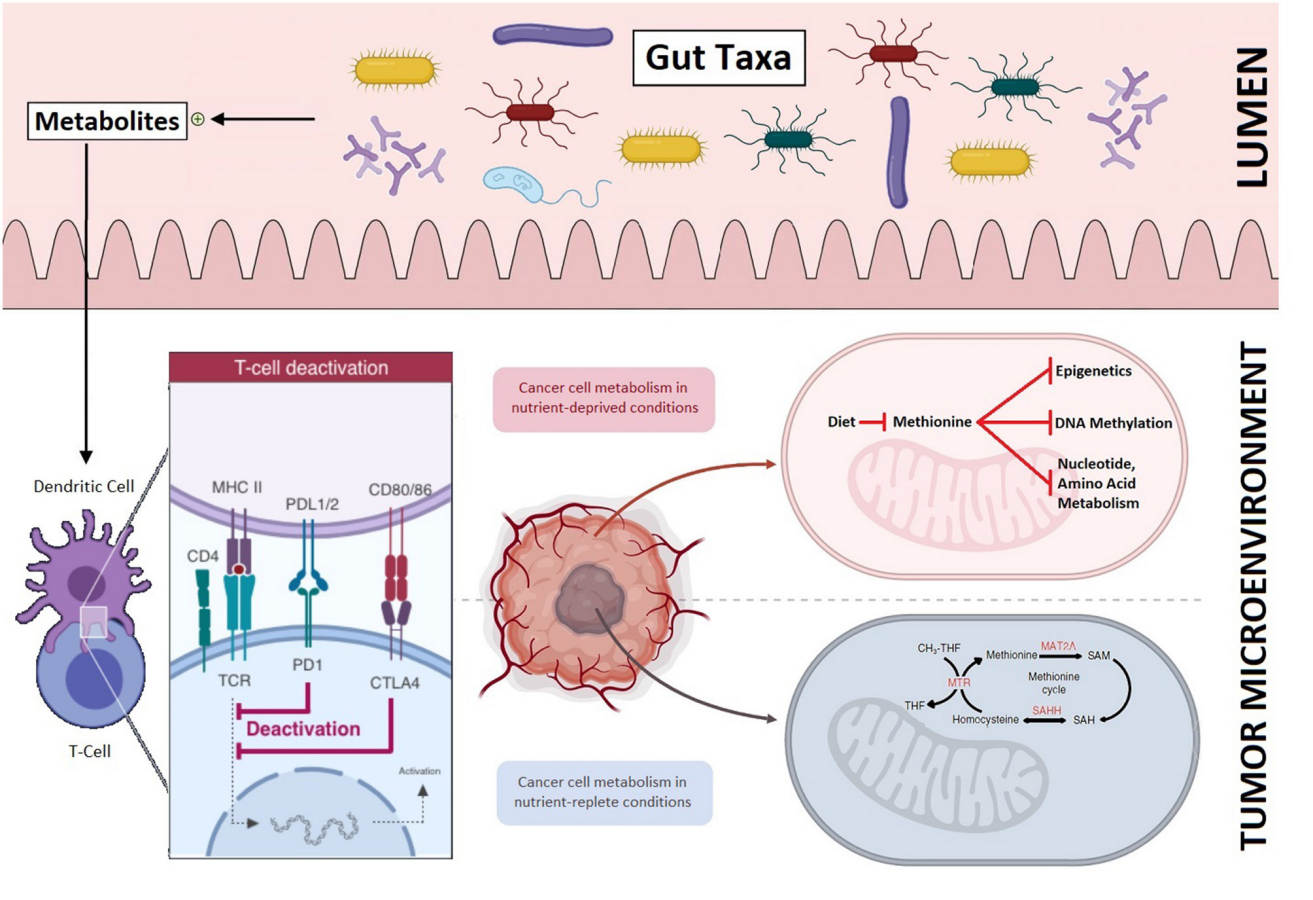
The role of methionine restriction in impeding tumor growth and its potential to improve outcomes in conjunction with gut microbiome manipulation: Resident gut taxa in the intestinal lumen produce metabolites **(top panel)** that direct T-cell differentiation and improve responses to immunotherapy **(bottom left)**. Differential tumor growth in methionine enriched and depleted tumor microenvironments **(bottom right)**.

In the era of harnessing genome editing to knockout tumorigenic (and immune inhibitory) proteins and sophisticated immune-engaging therapy, simple dietary interventions to achieve tumor control certainly holds similar promise. Strategic restriction of essential amino acids, in conjunction with chemotherapy, is being re-explored. The safety profile and optimal dosing regimen still need to be determined in large prospective trials. The intriguing question of whether methionine restriction could impart a similar metabolite-induced impact on immune-based cancer treatment also needs to be explored. Yet again, the key will be to bridge the gap between the bench and the bedside collaboratively.

## Author Contributions

MBA and MAA conceived of the idea and wrote the manuscript. MAA drew the figure. All authors contributed to the article and approved the submitted version.

## Conflict of Interest

The authors declare that the research was conducted in the absence of any commercial or financial relationships that could be construed as a potential conflict of interest.
